# Mediastinal Primary Synovial Sarcoma With Postoperative Pericardial Recurrence in a Previously Treated Angiofibroma

**DOI:** 10.7759/cureus.23014

**Published:** 2022-03-09

**Authors:** Yukari Kanemitsu, Naonori Inoue, Atsushi Sirakawa, Koichiro Mandai, Takuji Kawamura, Koji Uno

**Affiliations:** 1 Department of Gastroenterology, Kyoto Second Red Cross Hospital, Kyoto, JPN

**Keywords:** oncologic emergency, recurrence, cardiac tamponade, angiofibroma, synovial sarcoma

## Abstract

Synovial sarcoma is a malignant soft tissue tumor that often occurs near the limb joints. Here, we report a case of a patient with a synovial sarcoma that occurred in the mediastinum. The initial pathological diagnosis was suspected angiofibroma after surgical resection. After surgery, the tumor recurred in the pericardium and caused cardiac tamponade. Pericardial fenestration was performed and the patient was diagnosed with synovial sarcoma. The final diagnosis was the postoperative pericardial recurrence of the mediastinal synovial sarcoma. It is important to consider follow-up on the basis of the malignant tumor, especially if the disease is rare.

## Introduction

Synovial sarcoma often occurs near the limb joints. It rarely occurs in the mediastinum. The standard treatment is surgical resection. We report a case of a patient with synovial sarcoma that occurred in the mediastinum. Postoperative synovial sarcoma recurred in the pericardium, resulting in cardiac tamponade. The initial pathological diagnosis was angiosarcoma. Synovial sarcoma was diagnosed via pericardial fenestration for cardiac tamponade, and the initial pathological diagnosis was changed to synovial sarcoma. When the pathological diagnosis is a rare disease, it is necessary to follow up with the patient because a malignant tumor such as synovial sarcoma can occur.

## Case presentation

**Figure 1:**
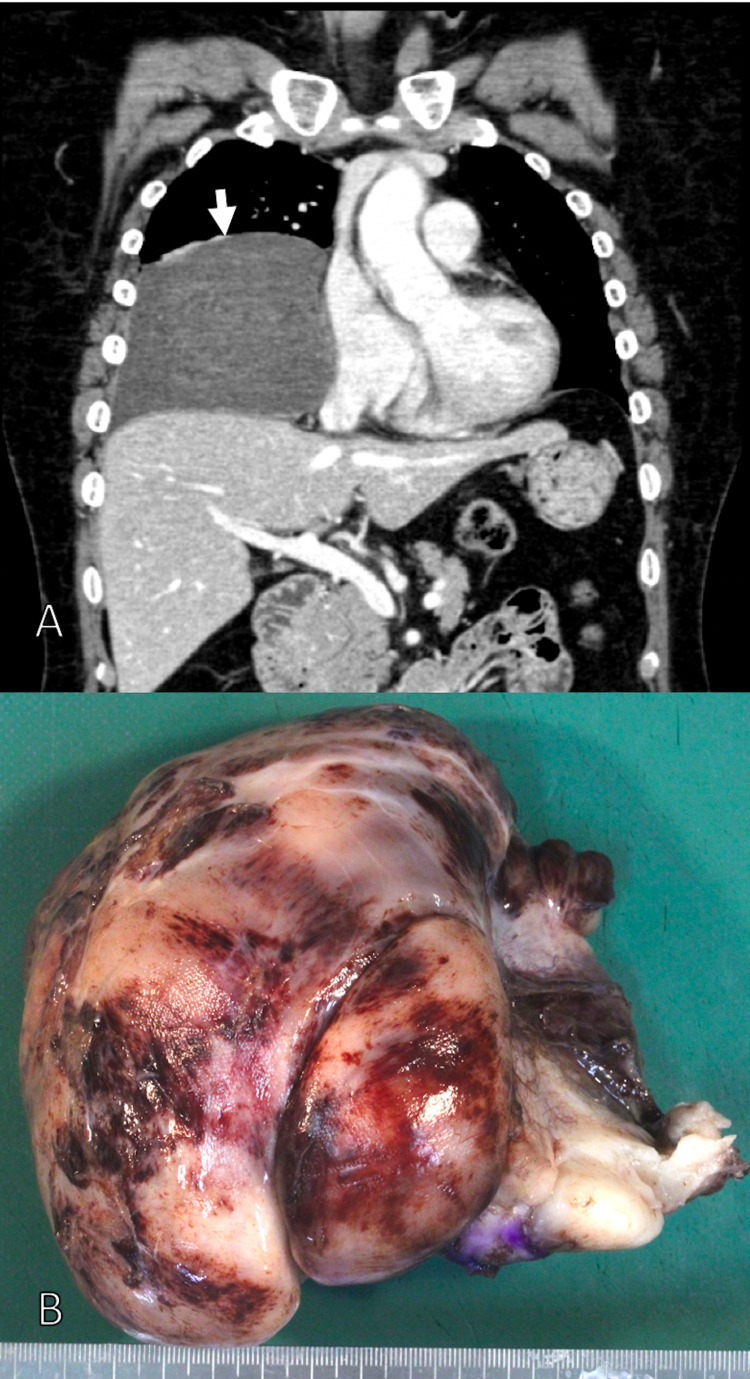
(A) Contrast-enhanced CT revealed tumor (arrows), which excluded the right diaphragm and lungs. (B) Macroscopic findings of the tumor.

This case involves a 59-year-old woman with a history of dyslipidemia and uterine fibroids. Breast magnetic resonance imaging (MRI) in October 2016 revealed a 3.5-cm mass in the posterior mediastinum (retrospective). She had an abdominal echo in October 2017, and a 13-cm mass was found in her mediastinum. The tumor could be confirmed by endoscopic ultrasonography from the esophagus. An endoscopic ultrasound-guided fine-needle aspiration biopsy of the esophagus was performed in November 2017. Spindle-shaped cells were present, but no diagnosis was made. MRI and computed tomography (CT) imaging findings showed that the tumor excluded the right diaphragm and lungs (Figure 1A), and surgery was indicated. Mediastinal tumor resection was performed in December 2017 (Figure 1B). The resected tumor had diffuse growth of spindle-shaped cells. A capillary network was found in the stroma, accompanied by hemorrhage. Soft tissue tumors such as angiofibroma, solitary fibrous tumor, and low-grade fibromyxoid sarcoma were identified. Immunostaining was positive only for the epithelial marker epithelial membrane antigen (EMA). Signal transducer and activator of transcription (STAT) 6 and mucin (MUC) 4 were negative. The pathological result was suspected angiofibroma. The patient then underwent CT follow-up every three to six months until June 2020 and no tumor recurrence was observed.

**Figure 2:**
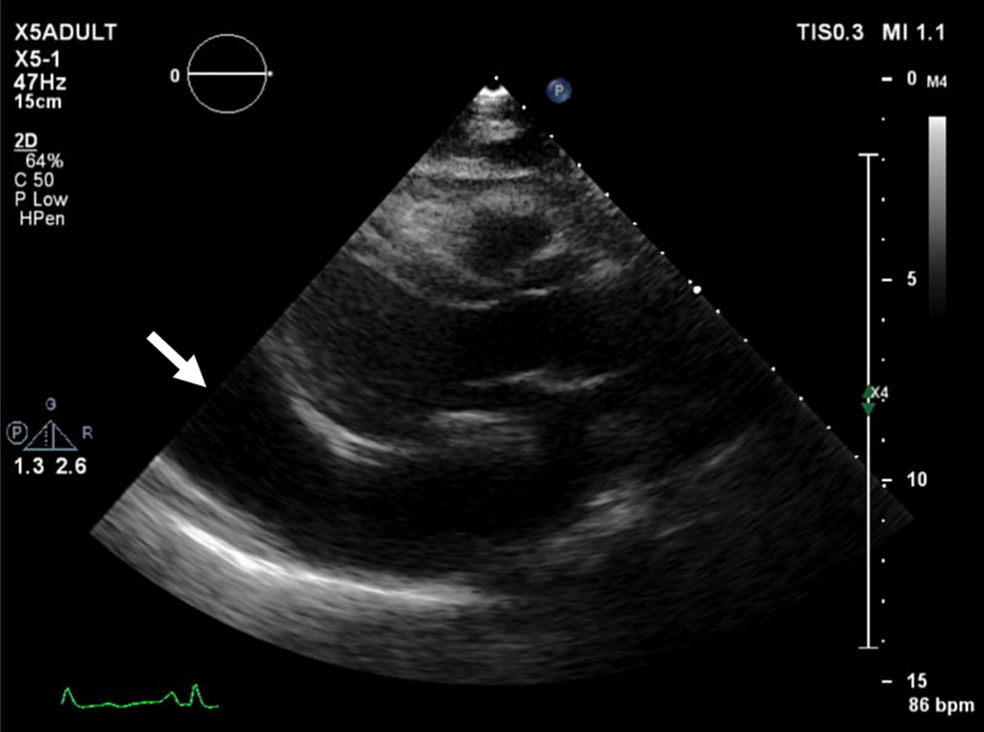
Two-dimensional echocardiography showed massive pericardial effusion (arrow) causing cardiac tamponade.

The patient then attended the hospital on September 8, 2020, with difficulty breathing. Her symptoms worsened in the recumbent position, indicating orthopnea. Echocardiography revealed pericardial fluid, and she was in a state of cardiac tamponade and heart failure (Figure 2).

**Figure 3:**
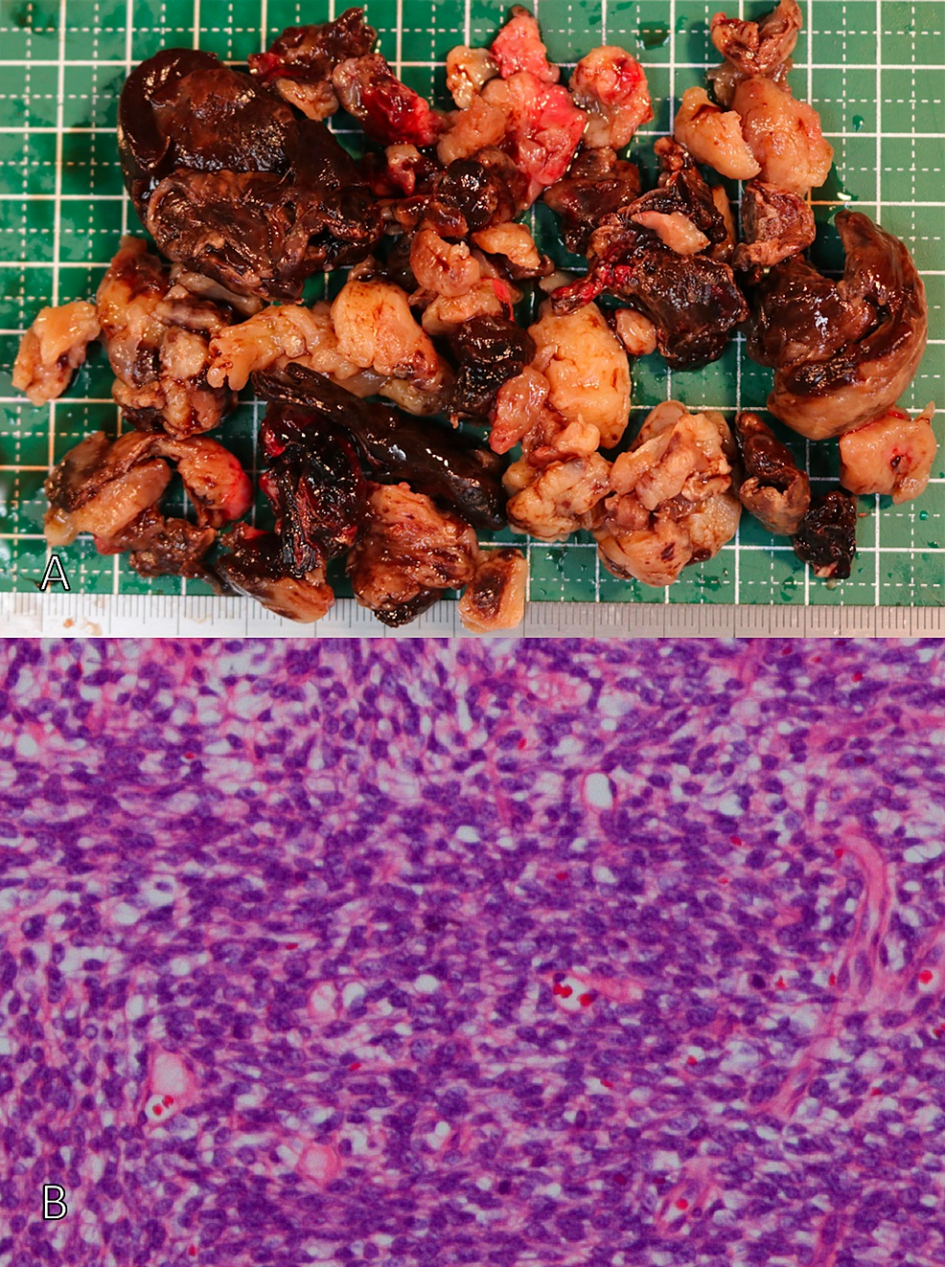
(A) Macroscopic findings of the tumor. (B) Hematoxylin and eosin-stained section revealed the tumor composed of spindle cells.

The patient was admitted to the hospital the same day. She underwent pericardiocentesis, but effective drainage was not attained. CT of her chest showed no change in the pericardial effusion. On day nine of illness, she underwent pericardial window surgery to remove the fluid from her pericardial sac (Figure 3A). Her tissue showed an increase in spindle-shaped cells (Figure 3B). The results of immunostaining were transducin-like enhancer of split (TLE) (+), pan-cytokeratin (+), and cluster of differentiation (CD) 34 (±). Genetic testing confirmed SYT-SSX2 fusion gene expression. Thus, she was diagnosed with synovial sarcoma. A mediastinal tumor specimen had been removed in 2017, and it was compared with a specimen removed from the intracardiac sac in 2020. The basic cell morphology was similar. Thus, the mediastinal tumor removed in 2017 was not considered to be a hemangiofibroma, but rather it was a synovial sarcoma. Therefore, this patient was diagnosed with mediastinal primary synovial sarcoma with postoperative pericardial recurrence.

Contrast-enhanced CT was performed on September 25, 2020, and the pericardial fluid had disappeared. The patient then underwent postoperative chemotherapy. The cardiologist advised that anthracycline-based drugs are difficult to administer considering cardiac function. Therefore, we decided to treat with pazopanib, which is a multityrosine kinase inhibitor. She started taking pazopanib on October 14, 2020. Hepatic disorder, which was thought to be caused by pazopanib, was observed, but it improved with a temporary drug suspension. Contrast-enhanced CT on December 15, 2020 showed a recurrent lesion in the mediastinum and a disseminated lesion in the bottom of the right lung. This was considered to be progressive disease (PD), and oral pazopanib administration was terminated. The patient was then re-admitted to the hospital due to the deterioration of her respiratory condition. She was transferred to another hospital for palliative treatment.

## Discussion

We report the case of a patient with synovial sarcoma that recurred in the pericardium and caused cardiac tamponade. If the pathological diagnosis is a rare disease, follow-up is required due to the possibility of a malignant tumor.

Malignant soft tissue tumors account for less than 1% of all malignant tumors. Among them, synovial sarcoma accounts for 5-10% of tumors, and they often occur in young adults and are most often reported in soft tissues near the limb joints [1]. Synovial sarcoma is prone to local recurrence and distant metastasis. The five-year survival rate is 30-50%, and it is a disease with a poor prognosis. Prognostic factors include tumor size, histological malignancy, stage, and incomplete resection [2]. The most important factor was reported to be incomplete tumor resection [3]. In this case, the tumor had entered the pericardium during tumor resection in 2017, and the pericardium was partially resected. The patient had pericardial recurrence 2.9 years after her initial surgery. There are multiple reports of synovial sarcoma originating from the pericardium [4-6]. If the pericardium is the primary tumor, cardiac tamponade can occur.

Cardiac tamponade that is caused by a tumor is an oncologic emergency that requires immediate treatment. Various reports showed that the incidence of malignant pericardial fluid ranges from 0.1% to 20%, and the most common primary tumor site is the lung [7]. Symptoms such as orthopnea, sweating, cold sensation, jugular vein distension, and loss of consciousness appear when cardiac tamponade occurs. Orthopnea was observed in this patient. Circulatory failure due to cardiac tamponade can be fatal, but appropriate treatment may result in dramatic improvement. In this case, the tumor was present in the pericardial sac, and pericardial resection was required to release the cardiac tamponade.

In addition to surgical resection, radiation therapy and drug therapy are used to treat synovial sarcoma [8]. This case involved postoperative pericardial recurrence, and drug therapy was selected as the treatment. Ifosfamide and doxorubicin are often used as drug therapies. Doxorubicin is difficult to use if heart failure is caused by cardiac tamponade, as in this case, because it has the side effect of cardiotoxicity. The multikinase inhibitor pazopanib has been shown to be effective against synovial sarcoma [9,10]. In this case, doxorubicin was not used for the abovementioned reasons, and treatment with pazopanib was selected. Trabectedin is also a useful drug for synovial sarcoma [11]. Further research to discover effective drugs for synovial sarcoma is required.

If the pathological diagnosis is a rare disease, the possibility of a malignant tumor should be considered and the patient should be followed-up. In this case, the postoperative pathological diagnosis was initially suspected to be angiofibroma, which is a relatively recently established disease [12,13]. Angiofibroma that develops in soft tissues is rare. It may be found on the abdominal wall, pelvis, or breast, but it is not common. In this case, the pathological diagnosis was suspected to be angiofibroma, but this was not confirmed. Therefore, after surgery, CT follow-up was performed every three to six months. However, there were no signs of recurrence until the onset of cardiac tamponade.

Differentiated diseases involving spindle-shaped cell sarcoma include fibrous tumor, malignant peripheral nerve sheath tumor, elevated cutaneous fibrosarcoma, leiomyosarcoma, and synovial sarcoma, and it can be difficult to distinguish between these sarcomas. The malignancy also varies, and it can be fatal depending on the recurrence location, as in this case. If a definitive diagnosis cannot be made, relatively frequent imaging follow-up should be considered. However, the recommended frequency has not been determined.

## Conclusions

In conclusion, we experienced the case of a patient with a mediastinal primary synovial sarcoma with postoperative pericardial recurrence in a previously treated angiofibroma. Postoperative synovial sarcoma caused cardiac tamponade when it recurred in the pericardium.
